# Economic value of HPC experience for new STEM professionals: Insights from STEM hiring managers

**DOI:** 10.3389/frma.2024.1462329

**Published:** 2025-01-22

**Authors:** Winona Snapp-Childs, Claudia M. Costa, Daniel Olds, Addison Snell, Julie A. Wernert, Craig A. Stewart

**Affiliations:** ^1^Pervasive Technology Institute, Office of the Vice President for Information Technology, Indiana University, Bloomington, IN, United States; ^2^Research Technologies Division, Office of the Vice President for Information Technology, Indiana University, Bloomington, IN, United States; ^3^Intersect 360 Research, Sunnyvale, CA, United States; ^4^Olds Research, Beaverton, OR, United States; ^5^Department of Computer Science, Luddy School of Informatics, Computing, and Engineering, Indiana University, Bloomington, IN, United States

**Keywords:** STEM, STEM careers, high performance computing, HPC, cyberinfrastructure, XSEDE, experiential learning, ROI

## Abstract

**Purpose:**

The purpose of this article is to investigate particular aspects of the STEM job market in the US. In particular, we ask: could the possession of high performance computing (HPC) skills enhance the chances of a person getting a job and/or increase starting salaries for people receiving an undergraduate or graduate degree and entering the technical workforce (rather than academia)? We also estimate the value to the US economy of practical experience offered to US students through training about HPC and the opportunity to use HPC systems funded by the National Science Foundation (NSF) and accessible nationally.

**Methods:**

Interviews and surveys of employers of graduates in STEM fields were used to gauge demand for STEM graduates with practical HPC experience and the salary increase that can be associated with the possession of such skills. We used data from the XSEDE project to determine how many undergraduate and graduate students it enabled to acquire practical proficiency with HPC.

**Results:**

People with such skills who had completed an undergraduate or graduate degree received an initial median hiring salary of approximately 7%–15% more than those with the same degrees who did not possess such skills. XSEDE added approximately $10 million or more per year to the US economy through the practical educational opportunities it offered.

**Discussion:**

Practical hands-on experience provided by the US federal government, as well as many universities and colleges in the US, holds value for students as they enter the workforce.

**Conclusion:**

Practical training in HPC during the course of undergraduate and graduate programs has the potential to produce positive individual labor market outcomes (i.e., salary boosts, signing bonuses) as well as to help address the shortage of STEM workers in the private sector of the US.

## 1 Introduction

The existence of a positive relationship between investment in innovation and societal economic benefits was posited at least as early as 1912 [recently reprinted in Schumpeter (2003)]. The existence of such a relationship has been supported by considerable evidence (National Academies of Sciences, Engineering, and Medicine, 2020) and is widely accepted within academia and government. A positive relationship between university-based innovation and economic benefits is strongly presented in Audretsch (2014). Winters (2014) shows that STEM (science, technology, engineering, and mathematics) graduates create significantly more economic benefit for society as a whole than non-STEM majors do, and that people majoring in a STEM discipline on average receive a higher economic yield on their education than students who major in non-STEM fields.

Despite the clear benefits to individuals of pursuing a major in a STEM field, the US faces a shortage of STEM workers, even though for employment in specific areas of academia and for some areas of science, there is an oversupply of new PhD recipients (Larson et al., 2014; Xue and Larson, 2015). Existing reports assert in strong terms that STEM professionals play a critically important role in both the US economy and global competitiveness (e.g., Aerospace Industries Association, American Association for the Advancement of Science, American Chemical Society, American Geophysical Union, American Physical Society, Consortium of Social Science Associations, Council on Competitiveness, Federation of American Societies for Experimental Biology, Institute of Electrical and Electronics Engineers USA and Semiconductor Industry Association, 2020). A report by the President's Council of Advisors on Science and Technology (2012) postulated that from 2015 to 2025 the US economy would need one million more STEM professionals than the US educational system was going to produce. A very recent report from the US Bureau of Labor Statistics indicates that as of 2022, there were 10,365,000 STEM jobs in the US, and it projects an additional 1,122,400 jobs by 2032 (US Dept of Labor, 2024). A clear indicator that the US is not developing and training its own talent in ways sufficient to fill demand for STEM jobs is that nearly half of current US STEM workers were born outside the US (Lehne, 2024).

Simultaneously, the relationship between an individual student and their future in a STEM field can be complex, leading to uncertainty and making the link between “there is a tremendous demand for STEM professionals in the US” and “I am therefore going to major in a STEM subject” tenuous for the individual. A recent longitudinal study of undergraduate students upheld all of the previously mentioned generalities while highlighting several other crucial factors, including geographic location, that influence the connection between a STEM major, employment, and salary (Lysenko and Wang, 2023). Taking a specific and longstanding example from one field, the production of astronomy PhDs has exceeded the demand for such experts in the academic market for years (Metcalfe, 2008). A recent National Science Foundation (NSF) report suggests that for PhDs in areas other than computer science *per se*, between 2/3 and 3/4 of new PhD recipients receive their degree without also possessing a firm job commitment (National Center for Science and Engineering Statistics National Science Foundation, 2021). In fact, one writer has even labeled the disparity between the production of new PhDs and the capacity of the academic market in the US as a crisis (Malloy et al., 2021). This overproduction of PhDs relative to demand within academia has unquestionably had negative effects within the academic job market, including an increase in postdoctoral positions strictly dependent on extramural funding (Cantwell and Taylor, 2015), the depression of salaries for postdoctoral researchers, and people spending very long periods of time in postdoctoral roles (Sarrico, 2022).

Many disciplines of science and engineering now make extensive use of high performance computing (HPC). This means that people at the undergraduate and graduate level may have significant experience with HPC by the time they receive their diplomas, and the possession of such skills may affect their job and career options. We present in this paper new data on the following questions:

- From the standpoint of organizations that hire STEM professionals and those analyzing the job market for STEM professionals:
  – Is there evidence of a particular need for individuals with skills in HPC?
  – How much additional salary are employers willing to offer to new degree holders who have practical experience in HPC programming as compared to those who do not?
- From the standpoint of a student considering undergraduate or graduate studies in a STEM field, what sort of demand exists for students with a STEM degree and practical experience in HPC in ways that are different from students with a STEM degree and without such practical experience?

The answers to the above questions can be of use to technology organizations, prospective and current students, and educational organizations. From the standpoint of technology organizations, the answers offer potentially useful insight regarding aspects of competition for talent among new holders of academic degrees. From the standpoint of prospective and current students in STEM fields, the answers to these questions can help inform perspectives on choice of career and course of study - and whether students should also focus on acquisition of practical experience with HPC. For educational institutions, which many people believe ought to be mindful of the relationship between academic studies and potential careers for students, this information may prove useful in devising both credit-bearing and non-credit-bearing learning activities for students in disciplines where the production of degree holders may exceed market needs for new degree holders within the specific discipline of their academic field(s) of study.

We pursued the above questions with considerable anecdotal evidence already in hand that this salary difference would turn out to be significant in statistical and practical terms. Investigation of the above topics leads naturally to questions about mechanisms for providing the training and resources that enable students to achieve practical competence with HPC. Such training and development of practical skills with HPC are typically not acquired through taking a for-credit course at a college or university. For 11 years, from 2011 to 2022, the NSF funded a project called XSEDE—the eXtreme Science and Engineering Discovery Environment—to provide support for a suite of advanced cyberinfrastructure systems (supercomputers, HPC clusters, clouds, data storage systems, and visualization systems) to the national academic research community. Support for such systems included a variety of functions such as management of applications through a peer-review merit system, offering them at no cost to the US research community. Additionally, the project involved accounts management, security measures, support services (including consulting and programming support), training, and outreach activities. These services are described in detail in Towns et al. (2014) and Stewart et al. (2023). The training program offered by XSEDE was designed to provide students with sufficient knowledge to effectively use and program HPC systems. This program enabled students to run, modify, and, when necessary, even develop HPC software from scratch. With the help of this educational program, students were enabled to fulfill educational objectives and also acquire practical competence in the use of HPC resources. The NSF-funded computational resources provided by XSEDE, along with the support and allocation facilitated by XSEDE, enabled students to use HPC systems for their research projects. As a result, many students were able to attain a practical level of proficiency in using HPC systems. However, there is very little systematically acquired and quantitative information available regarding the value of practical educational activities such as this training of students, particularly when the training is offered through means other than those that accrue formal college or university course credits. This leads to three additional questions addressed in this paper:

- What value did XSEDE deliver to students who took advantage of XSEDE training programs and used XSEDE-supported HPC systems to achieve a level of practical competence in the use of such advanced computational systems?
- What value did XSEDE deliver to the US economy?
- From the standpoint of leaders of educational institutions and policymakers at state and federal levels, is it economically beneficial to invest in experiential learning opportunities to increase the supply of STEM professionals entering the job market with HPC practical experience?

## 2 Background and prior work

### 2.1 XSEDE

XSEDE and its accomplishments are described at great length in Towns et al. (2014), XSEDE (2023), and Stewart et al. (2023). Regarding NSF support, what is commonly referred to as XSEDE was supported by two different NSF grant awards: XSEDE and XSEDE2. Funding through the XSEDE2 grant award supported services offered from 2016 to 2022; this is the time period for which we analyze the economic value of XSEDE training programs. For simplicity, we will refer to this resource as XSEDE. The following quote describes some of the key aspects of XSEDE and its services [from Stewart et al. (2023)]:

…* the NSF has funded two related but distinct types of cyberinfrastructure services [from 2011 to 2022]: XSEDE, serving as a general “front door” and support mechanism; and service providers (SPs), which operate cyberinfrastructure resources such as supercomputers, high performance computing (HPC) clusters, storage systems, and visualization systems. The resources operated by these SPs are available to the US research community for purposes of non-classified and non-commercial research. ... [Some] services offered by XSEDE and by SPs are allocated through a process in which principal investigators (PIs) apply for resources such as computer time or expert consultant time. ..*.

- *XSEDE supported a total of 15 supercomputers and HPC clusters and one cloud system with an aggregate processing capability of 28.6 petaFLOPS ... (One petaFLOPS is one quadrillion floating-point mathematical operations per second.)*
- *XSEDE employed a total of 212 individuals for a total full-time equivalent (FTE) of 91.8 individuals*.
- *XSEDE directly supported more than 11,000 researchers and students, including individuals in every state in the US, the District of Columbia, Puerto Rico, Guam, and the US Virgin Islands. ... More than 18,000 other individual users ...[were] supported indirectly via Science Gateways, web-based interfaces customized for particular analysis and simulation tasks*.

The return on federal investment in XSEDE has already been investigated in detail in several regards. XSEDE provided services to the US academic research community at somewhere between one-half and one-third the cost that would have been required to purchase such services on the commercial market in the US - a return on investment (ROI) on US federal government investment of two to three (Stewart et al., 2023). More recently, Snapp-Childs et al. (2024) have argued plausibly that the value delivered to the US as a result of XSEDE's contributions to the economy and quality of life in the US exceeded the cost of XSEDE to the US federal government.

### 2.2 XSEDE training and education activities

The workforce development mission of XSEDE was “to provide a continuum of learning resources and services designed to address the needs and requirements of researchers, educators, developers, integrators, and students utilizing advanced digital resources.” From 2016 to 2022, XSEDE offered periodic HPC workshops to teach practical HPC programming techniques to the research computing community. These workshops were available in various formats, including live web sessions, prerecorded web content, written materials, and in-person classes. This included 146 different training courses and a YouTube channel providing 30 lectures, which are still available for viewing (XSEDE, 2023). These training events reached nearly 9,000 attendees over the course of the 2017-2022 time period studied. A detailed longitudinal study of this program and evaluation of its effectiveness as a training effort is provided in Miles et al. (2023).

## 3 Methods

In this paper, our focus is on STEM degrees but specifically excluding degrees in computer science, computer engineering, and related fields. We make this exclusion because these majors are currently a special case due to the tremendous market demand for individuals with skills in the currently rapidly growing field of artificial intelligence (AI). Considering the current value of the US dollar and cost of living, a computer science degree with specialization in AI may hold an exceptionally high value at this moment, possibly even an all-time high.

### 3.1 Interviews and surveys of private industry

Companies in the private sector that make use of HPC often view much (perhaps most) information about their activities as competitively sensitive. In order to obtain thorough and frank information about employee needs and hiring practices, Indiana University contracted Intersect360 Research to conduct surveys and interviews. Intersect360 Research (Intersect360, 2024) is an industry analysis firm that specializes in tracking and predicting trends across high-performance data center markets, such as HPC, AI, cloud computing, and big data analytics. Intersect360 Research surveys, forecasts, and analysis are trusted by government agencies, policymakers, and leading companies in the HPC and AI technology landscape, who rely on Intersect360 Research analysis in shaping their strategies. Intersect360 Research has been in existence for more than 18 years and during that time has assembled a list of individuals and organizations that make use of HPC technologies, spanning public- and private-sector organizations worldwide, which it surveys regularly on topics such as budget trends, technology usage, and outlook. Intersect360 Research maintains a database of approximately 1,000 organizations that make use of HPC users worldwide and/or are involved in STEM fields that it regularly surveys as part of its market analysis research. The advisory board for an organization called HALO (HPC-AI Leadership Organization) includes leaders of the kinds of public- and private-sector organizations typically surveyed in Intersect360 market studies (HALO, 2024). Intersect360 surveyed and interviewed representatives of hundreds of the companies in its database for the purpose of obtaining information for this study.

We began data collection regarding practical experience with HPC with a series of 27 telephone interviews to get a sense of hiring managers' perspectives on this issue. The purpose of these interviews was primarily to get enough information about the perspectives of hiring managers to create an effective online survey. The interviewees were not chosen randomly but were instead chosen on the basis of two criteria: to represent a cross-section of the concerns of those who hire staff with HPC expertise and to get the perspectives of hiring managers willing to participate in an extended interview. These interviews yielded many great insights and quotes. Basic analysis of the text notes from these interviews was done with the qualitative analysis software package nVivo version 15 (Lumivero, 2024). Based on these interviews and the research objectives of this study, we created an online survey form (with a modest amount of skip logic built into it so that respondents did not need to deal with questions irrelevant to their particular circumstances). The survey instrument itself is available online at Snell et al. (2023). Full results are available online at Snapp-Childs et al. (2023a). The survey instrument was approved in advance by the Indiana University Institutional Review Board. A total of 325 surveys were completed, all from US-based organizations that had previously participated in Intersect360 HPC market surveys. The respondents included 16 academic organizations, 255 commercial organizations, 52 government organizations, and two organizations which did not specify an organization type.

### 3.2 Usage of XSEDE resources by undergraduate and graduate students

Information on usage of XSEDE-supported systems is stored in the XSEDE Metrics on Demand (XDMoD) system (Furlani et al., 2013). The data stored include an entry for each computing job executed (interactively or in batch form) by each account holder. Information stored also includes the aggregate number of jobs run, an aggregate account of computer resources consumed, the aggregate number of days on which a person ran a computer job, and a calculated average number of jobs per individual and average number of days active per individual. The averages do suggest (very coarsely) different behaviors by the different groups. The entries for each individual account holder are numbered for record keeping but anonymized.

Using this information, we estimated the number of students who had sufficiently used XSEDE-supported HPC systems to indicate the acquisition of a practical level of skill. We based our estimate on an analysis of usage patterns and distribution, with particular emphasis on the “days active” metric. This metric appeared to be the most reliable indicator of a student's achievement in using HPC systems to a practical extent.

### 3.3 Value of practical experience in the use of HPC enabled by XSEDE

To determine the economic value to the US economy of the training provided by XSEDE, we followed the basic methodology laid out in Stewart et al. (2023). Day and Martinez (2021) provide recent average salaries for new hires in STEM fields other than computer science. We used usage data and other XSEDE records to estimate the number of students who, during the course of the XSEDE project, received a good practical understanding of HPC before completing their degree and entering the job market. For these students, based on survey data collected as part of this research, we used three different measures of percentage salary increase to provide low- and high-end estimates of economic impact, along with what we believe to be a best overall estimate that lies between the two. These estimates of economic impact were calculated as shown below:


$$\begin{array}{l} Economic\;Impact\;=\;\left(average\;starting\;annual\;salary\right)\\ \;*(number\;of\;students\;who\;achieved\\ \;practical\;HPC\;skills\;via\;XSEDE)\\ \;*\left(salary\;raise\;difference\right) \end{array}$$

When calculating economic value, these computations were done separately for undergraduate and graduate students and then summed. We used a variety of different figures for estimating the number of students who have used XSEDE HPC resources to an extent that constitutes practical experience with HPC, and a number of different central measures of raise, in order to provide some sense of potential variability and sensitivity to particular elements of these calculations. We assume that the economic value created by XSEDE, represented in a salary differential, persists for only one year for two reasons. Firstly, it is based on the idea that a person hired into a job without practical HPC skills would likely acquire those skills on the job in a year. Secondly, this approach is conservative and helps avoid potentially overestimating the total economic value of XSEDE. Total economic value was calculated separately for undergraduate and graduate students, and then summed to obtain a cumulative value over the span of the six years studied. Finally, an average value per year was calculated.

## 4 Results

In this paper we focus on a subset of the responses most relevant to career and training planning for potential STEM majors, institutions of higher education educating and training STEM majors, and those who set organizational and public policy relevant to the STEM workforce in the US. Data on the initial set of interviews, including characteristics of the organizations that were subjects of interviews (conducted with hiring managers of these organizations) and the text of key responses by the interviewees, are available at Snapp-Childs et al. (2023a). The screen images of the online survey form are available online at Snell et al. (2023). The full dataset of online survey responses is online at Snapp-Childs et al. (2023b). A dataset of statistics regarding undergraduate student and graduate student use of XSEDE resources for the time period studied is available online at Hart and Towns (2024).

### 4.1 Data sources

Organizations that rely on HPC as a core part of their business are traditionally very careful with information that is potentially sensitive. By partnering with Intersect360, we were able to gather comprehensive data from those organizations. Although our survey did not follow a random sampling approach, we received excellent responses and obtained a representative collection of feedback. We believe that this trade-off, focusing on requesting information from organizations that trusted and would respond to questions from Intersect360, in order to get frank responses, was reasonable and justified as compared to a random sampling approach for this study. This study has no obvious precedent that we can find in the literature related to the US job market and is thus in many ways an early investigation rather than a final analysis of the current situation in the STEM job market.

Table 1 summarizes the market sectors represented among interviewees and respondents to online surveys. Initial interviews were held with 27 individuals, each of whom was employed as a hiring manager at an organization that operates in some sort of STEM field. These interviews were largely unstructured and were conducted primarily for the purpose of obtaining enough information to construct a useful online survey form.

**Table 1:** Respondents to online survey and participants in interviews according to market sector.

**Market sector**	**Interviews**	**Online survey**
Bio-sciences (pharmaceuticals, genomics, medical devices, etc.)	29.6%	7.9%
Electronics (semiconductors, electronic components, etc.)	7.4%	7.1%
Energy (oil/gas exploration, alternative energy)	18.5%	7.9%
Financial services, including insurance	0.0	6.7%
IT and computing (processor manufacturers, OEMs, software developers, IT services, etc.)	3.7%	16.1%
Manufacturing, automotive and aerospace, industrial	18.5%	15.7%
Manufacturing, consumer products	7.4%	24.8%
Media/entertainment	0.0	0.8%
Other manufacturing, including chemical engineering	3.7%	2.4%
Professional services (engineering consulting, cloud provider, government research, etc.)	7.4%	9.1%
Telecommunications	0.0	1.2%
Other	3.7%	0.4%

A total of 325 hiring managers were invited to complete our online survey. No more than one person per organization was invited to participate, and each invited participant did at least as much as filling out the early portions of the survey. Unsurprisingly, with what was a fairly long survey, some invitees did not complete the full survey.

### 4.2 Analysis of interview data

#### 4.2.1 Quantitative analyses

Initial interviews with hiring managers in organizations working in areas related to STEM fields were largely unstructured. An exception was the following specific question: “For your organization, in non-IT scientific and engineering, please let us know if the candidates below would be more valuable in terms of starting salary. What offer would your organization make to the candidates below?” [followed by a list of candidate characteristics indicating HPC knowledge/skills]. The responses are tabulated in Table 2.

**Table 2:** Responses to whether their organization offers higher starting salaries due to HPC experience.

**Response**	**Unqualified yes/no**
Yes	10
No	4

#### 4.2.2 Qualitative analyses

The interviews with hiring managers in STEM fields consistently upheld two ideas commonly discussed in reports about HPC, the job market, and the STEM economy of the US: practical experience in use of HPC systems is highly valued, and organizations that wish to hire employees with such skills find it hard to fill all of their open positions. The following quotes are examples of the kind of commentary offered by hiring managers during interviews:

- *When it comes to engineering, they've got a backlog of excessive workload that they can't satisfy, so they're looking for someone with specific skills that could fill that backlog of projects*.
- *If you've got any experience in parallel computing of some kind, it's a real asset. It's not something that a lot of universities have courses in, and so anywhere people can pick it up, … anybody who's kind of played with that at all, tends to have really valuable skills that we're looking for*.
- *We have various salary grades. [New hires] come in at an entry-level engineering salary grade. If you have a master's degree, you've probably come in bumped up into the next salary grade. Now these salary grades are ranges, and we have this nomenclature that the midpoint of the salary is called 100%. The entry point is 80%. And the top-out is 120%. And so usually, they want to hire someone to be somewhere between the 80% and 100% point. And anything that goes over that so-called “midpoint” of 100% would require some HR approval. … So Candidate A [who has no relevant experience], maybe they're at the 80% point. Then [Candidate B, with HPC-specific education but not practical experience] would then be at the 90% point, and [Candidate C with HPC-specific education as well as some practical experience] would probably be at the 100% point, based on where our current general guidelines are*.
- *...can think of a couple of recent hires that were very appealing because they had a strong computational background, … who have been like, system admins at school, as work-study, have done some significant HPC-type coursework, or maybe had an internship or a part-time job that's involved in computation*.
- *I might have to go up ten percent, five to ten percent, to get that person with some additional practical [technical computing] experience*.

Because the purpose of conducting these interviews was to obtain information that would help us design an effective survey instrument, the interviews were conducted less formally than would be the case in other circumstances. Also, in order to facilitate frank exchanges, we did not record the interviews (which were conducted over telephone or Zoom). As a result, the data that we have from the interviews are notes by interviewers rather than full text, so the results of these preliminary discussions are not suitable as input data for formal qualitative analysis. Interviewees were told of the general purpose of our study, and then simply asked to comment. There was one question that interviewers always did ask, about whether or not a new employee with classroom or practical experience with HPC might receive a higher starting salary.

Table 3 tallies the themes mentioned by interviewees in the mostly unstructured interviews.

**Table 3:** Themes discussed by interviewees in the mostly unstructured initial interviews.

**Theme**	**Interviewees**
Importance of technical computing	17
Communication skills and EQ (Emotional Quotient)	10
Level of education is important in hiring decisions	10
Relevant experience is important in hiring decisions	8
Increasing need for STEM professionals	5
Technical computing has a positive impact on long-term career path	8
Teamwork and initiative are important	3

Some specific comment in response to the question about whether HPC experience resulted in a difference in starting salaries was made by 14 interviewees. Out of these, 10 indicated that HPC experience is cause for a higher starting salary. The remaining four indicated that HPC experience was not associated with a higher starting salary, but there were clear indications from those respondents that the lack of a higher starting salary is imposed by organizational policies. There was some indication that a lack of a starting salary differential was more common at smaller organizations.

### 4.3 Surveys of hiring managers

#### 4.3.1 Quantitative analyses—Need for HPC skills, factors in hiring

Among the hiring managers who responded to the survey, 143 of 325 total respondents indicated that the organizations they represented were currently using some form of HPC (involving computing clusters or supercomputers). The results presented below are based on those survey responses.

The following tables present quantitative summaries of the most important questions on the topic of HPC and hiring. Table 4 shows that 96% of respondents “agree” or “strongly agree” that the need for employees with scientific and engineering skills is increasing. Further, 61% say their company has difficulty hiring new scientific and engineering personnel. Table 5 indicates that level of education is a strong factor in hiring decisions. Table 6 shows that 89% of hiring managers “agree” or “strongly agree” that educational experience with HPC is an important factor in hiring decisions.

**Table 4:** Survey responses from HPC-using firms regarding need for staff and issues in retention.

**Question**	**Strongly disagree**	**Disagree**	**Neutral**	**Agree**	**Strongly agree**
Need for scientific and engineering employees is increasing	0%	0%	3%	43%	53%
Have a problem hiring new scientific and engineering personnel	6%	11%	21%	34%	27%

**Table 5:** Survey responses from HPC-using firms on their top five hiring decisions factors.

**Question**	**No influence**	**Some influence**	**Major influence**
Level of education (bachelor's, master's, etc.)	2%	17%	81%
Work experience in relevant field, including internships	3%	20%	77%
Education or experience with computational science or engineering	8%	31%	61%
Demonstration of teamwork, ability to work in groups	11%	35%	54%
Examples of initiative beyond normal coursework	13%	37%	50%

**Table 6:** Survey responses on hiring new staff with HPC education or experience (HPC EE).

**Question**	**Strongly disagree**	**Disagree**	**Neutral**	**Agree**	**Strongly agree**
We seek to hire scientists or engineers with HPC EE	0%	2%	4%	51%	43%
HPC EE is a factor in selecting the best candidate	0%	2%	8%	49%	41%
HPC EE is a factor in determining starting salary or compensation	0%	4%	7%	53%	36%
HPC EE is a factor in long-term career advancement	0%	2%	9%	48%	41%

Survey responses also indicated a strong importance for the current role of HPC: 1% of respondents indicated that HPC was not currently important; 7% indicated that it was important; 92% indicated that it was currently very important. The importance of HPC is expected to increase significantly in the next five years compared to the present. Respondents rated the role of HPC five years from now, in their opinion, as being: less important, 2%; same importance, 30%; more important, 66%; not sure, 2%.

#### 4.3.2 Quantitative analyses—Starting salary and HPC experience

Table 7 below shows results of survey questions about how much of a salary increase, in percentage terms, would be given to a new hire with HPC practical experience as opposed to one with otherwise similar skills but lacking HPC expertise. This table provides simple measures of the central tendencies expressed for the entire respondent pool and for the subset (just over half) of respondents who indicated that their company would indeed offer some sort of increased salary for new employees with HPC skills.

**Table 7:** Salary increase differential for new hires with practical HPC experience^*^.

**Measure**	**All respondents**	**Only respondents with salary differential**
Mean % increase (+/− 1 SE)	7.64% (+/− 0.6%)	15.2% (+/− 0.9%)
Median % increase	2.0%	10.0%
Range of % increase	0% - 70%	2% - 70%
% of respondents indicating no additional pay for HPC experience	49.7%	–

^*^To test if the median salary percentage was above zero, a one-sample Wilcoxon signed rank test was used (p < 0.001).

It seems useful to break down central tendencies by category market sector and organization size. However, the data are not generated as a random sample of any sort of recognizable population. Rather, the data were selected by the rule “organizations that recognize and trust the Intersect360 consulting firm and deal in STEM areas.” In such a situation, the median may be the most informative measure of central tendency, since it is less likely than a mean to be strongly biased by a lack of randomness in the data set collection. Table 8 shows median percentage raises for responses from organizations that report that they make use of HPC, broken down by organizational size and market sector. The overall median response was 5%. The most common response (mode) was 0%, followed by 5%.

**Table 8:** Median salary raises by market sector and organizational size, only for HPC-using firms.

**Organization size**	**Very small firms 1-100**	**Small firms 101-1,000**	**Medium firms 1,001-10,000**	**Large firms 10,001-100,000**	**Very large firms 100,001 or more**
Academic, not-for-profit	0% (1)	0% (7)	0% (7)	10% (1)	ND
Bio-sciences	20% (1)	0% (7)	5% (5)	5% (7)	ND
Electronics	ND	32.5% (2)	0% (14)	15% (1)	5% (1)
Energy	0% (1)	6.5% (4)	0% (9)	7.5% (6)	ND
Financial services	ND	17.5% (4)	0% (11)	30% (1)	10% (1)
Government	5% (5)	5% (16)	5% (18)	0% (7)	0% (6)
IT provider	0% (3)	5% (11)	5% (23)	5.5% (4)	ND
Manufacturing, auto & aerospace	0% (5)	5% (13)	2.5% (8)	0% (9)	10% (5)
Manufacturing, consumer	0% (9)	7% (20)	0% (31)	10% (3)	ND
Manufacturing, other	0% (1)	0% (3)	5% (2)	ND	ND
Media/entertainment	ND	ND	ND	ND	ND
Professional services	2% (7)	5% (5)	2.5% (6)	2.5% (4)	0% (1)
Telecommunications	ND	ND	ND	ND	ND
Other	ND	10% (3)	0% (5)	ND	ND

Data shown as: median % raise (number of respondents per cell)^*^. ^*^ND in a cell indicates “no data”—either there were no firms invited for that combination of market sector and organizational size, or some were invited but indicated no HPC use.

#### 4.3.3 Qualitative analyses of responses to open-ended questions

**Figure 1:**
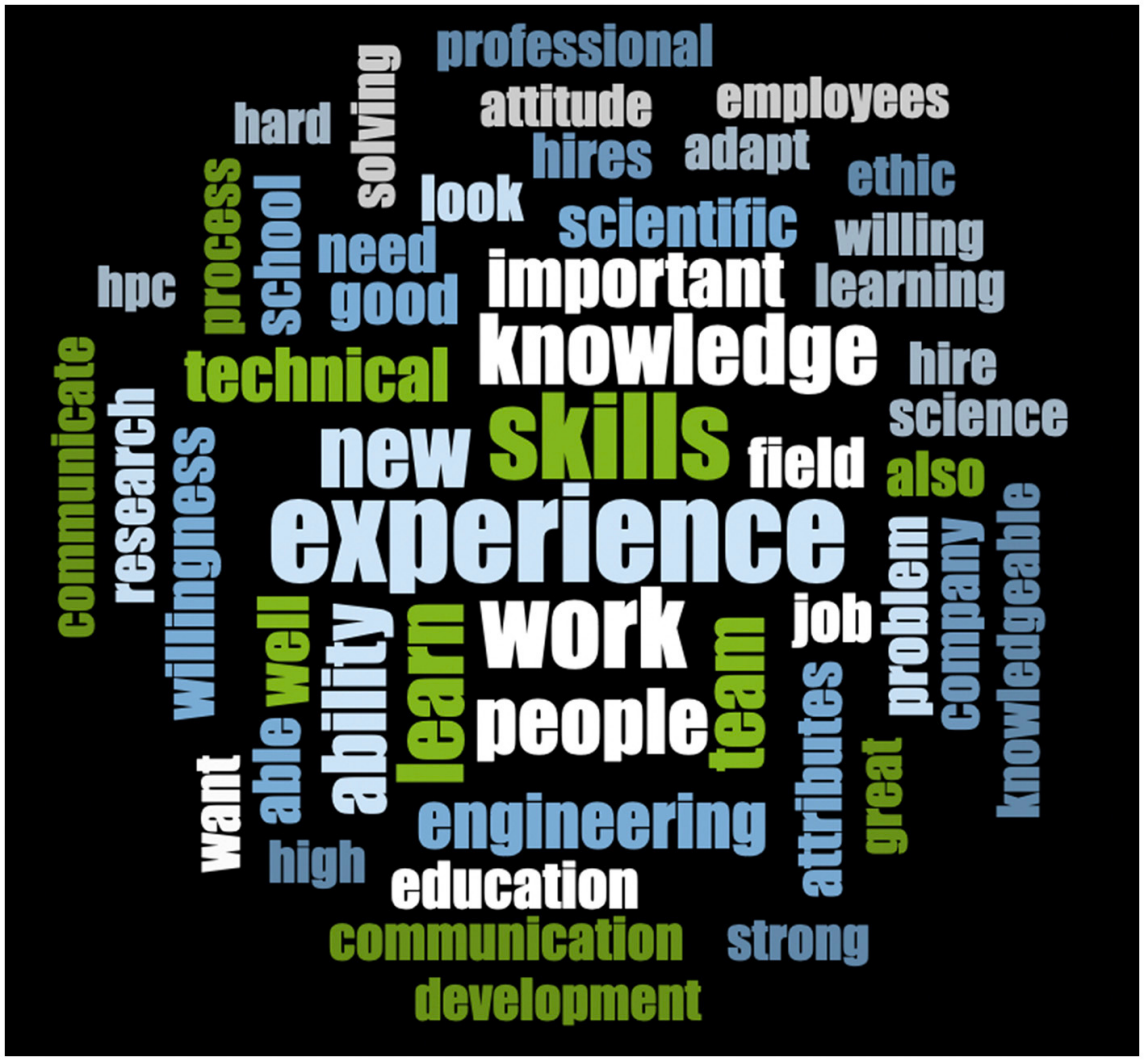
Word cloud—responses to the question “What attributes are most important for new hires in scientific/engineering fields?” using word length up to 2, showing 50 most frequent.

**Figure 2:**
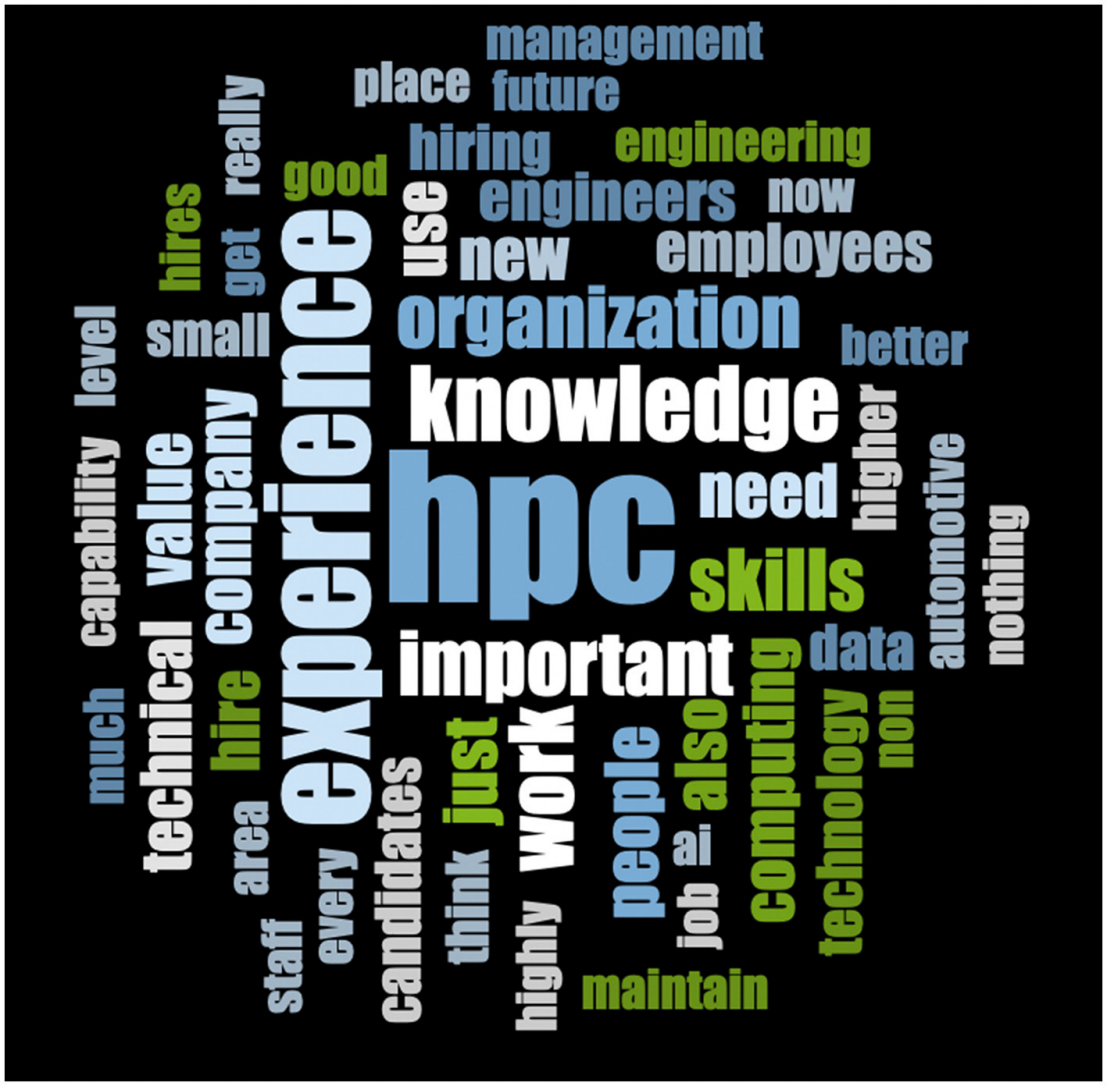
Word cloud—responses to the question “What attributes are most important for new hires in scientific/engineering word cloud—responses to the question “Open-ended question: Other thoughts?” using word length up to 2, showing 50 most frequent.

The online survey included two open-ended qualitative questions. The text of the responses to these questions was analyzed with nVivo version 15. Most of the responses were a single sentence or a phrase, which in retrospect is both what the layout of the survey encouraged and what one might expect near the end of a long survey. Given the fairly limited set of text - just a few thousand words - qualitative analysis revealed nothing beyond what one can see displayed in a simple word cloud. Word clouds (Figures 1, 2) were generated based on responses to the two qualitative questions asked in the online survey, using word length up to 2 and showing the 50 most frequent results. Figure 1 shows the word cloud for the first open-ended question, which was: “What attributes are most important for new hires in scientific/engineering fields?” Figure 2 shows a similarly constructed word cloud for the second open-ended question, which was simply: “Other thoughts?” Note that these questions were asked after dozens of questions about HPC had already been asked. In responses to the first question, HPC *per se* is mentioned less than might have been expected; respondents might justifiably have thought that they had already said quite enough about HPC. In responses to the last and completely open question, by contrast, HPC is the most common word.

There is relatively little in the shorter text corpus that was not already mentioned in some form in the quotes from the interviews. Here is a small and representative sample of the responses to the open-ended questions where hiring managers are commenting about the importance of HPC generally and/or the value of new hires with HPC experience:

- *very important for the future defense of the country*
- *we value them immensely because they are an important part of our manufacturing process*
- *this is a skill you must have when interviewing for a job*
- *this is a key area for the future growth of our enterprise and the success of our hires is crucial to its proper function*

### 4.4 Usage of XSEDE resources by undergraduate and graduate students

**Figure 3:**
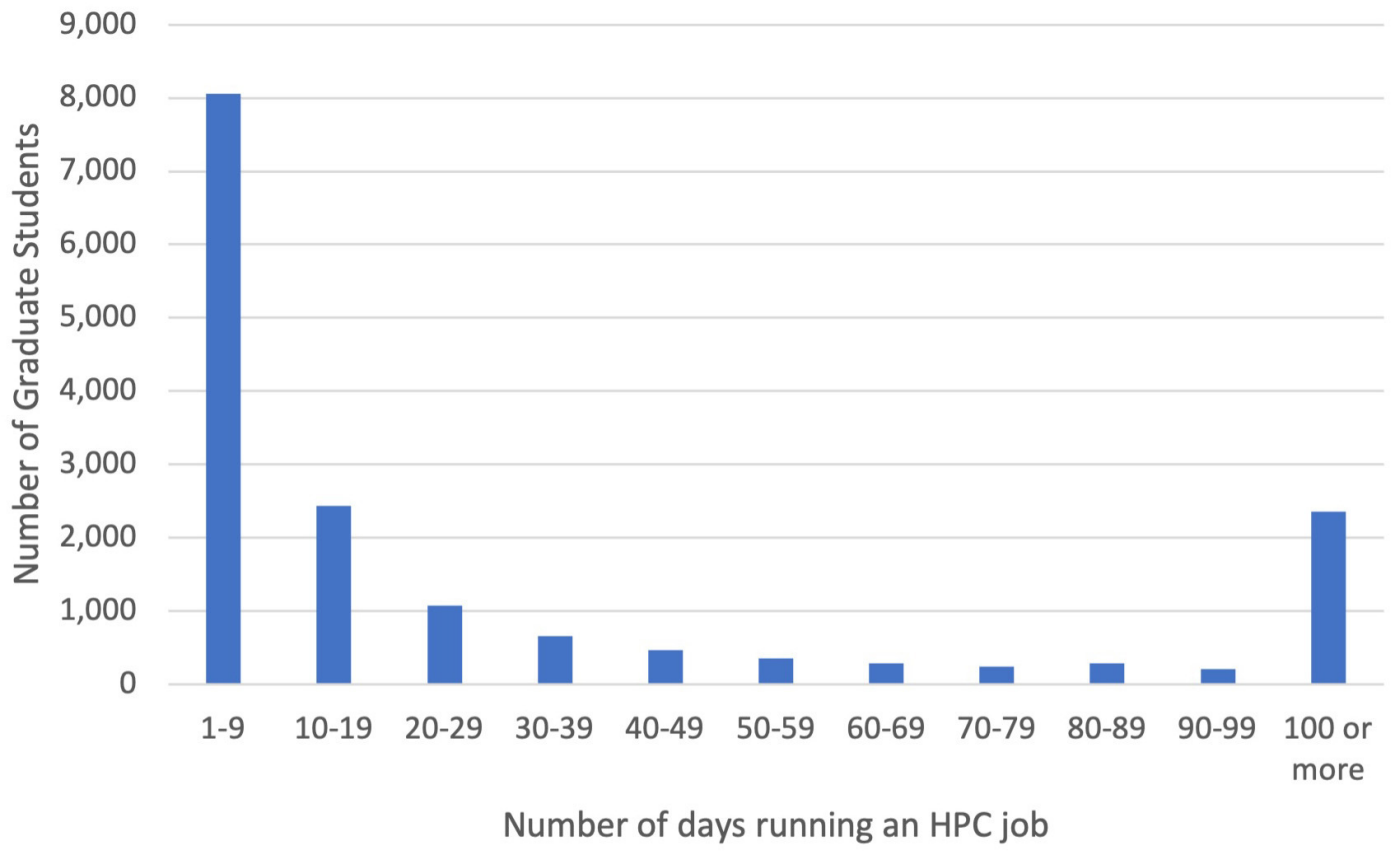
Distribution of number of days on which graduate students ran at least one HPC job on an XSEDE-supported resource between 2016 and 2022.

**Figure 4:**
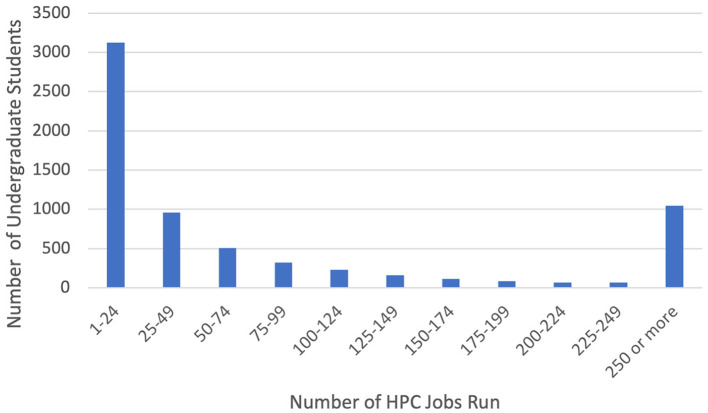
Distribution of number of HPC jobs run by undergraduate students using XSEDE-supported resources between 2016 and 2022.

The XSEDE accounting system records a number of statistics on a per-user basis, including total number of jobs run, total CPU hours used, date, and time jobs were run. From this it is possible to calculate the number of days on which any student ran at least one job as well as the total number of jobs run by a student. Figure 3 shows the distribution of number of days a graduate student ran a job ranging from 1 to 100 or more, grouped in 10s. The distribution of days on which an undergraduate student ran a job is qualitatively similar (Figure 4), although as one would expect, they run jobs on fewer days overall than graduate students. Both distributions show a very clear pattern: a very large number of students take some sort of introductory class such as a webinar or one-day tutorial, run one to a few jobs, and then never use XSEDE-supported HPC systems again. Partly because of this, and partly because a small minority of students executed a significant number of jobs on numerous days, the distribution of days on which students ran a job is highly skewed. Table 9 shows the basic descriptive statistics for HPC jobs on XSEDE resources run by undergraduate and graduate students.

**Table 9:** Usage of XSEDE-supported HPC systems for 2016–2022.

**Category**	**Undergraduates**	**Graduates**
Total student user count	6,684	16,415
Median jobs	29	49
Average jobs	431	1,967
Minimum jobs	1	1
Maximum jobs	214,324	1,036,898
Average days active	21	53
Median days active	7	10
Minimum days active	1	1
Maximum days active	911	2,494

A next question is then “what level of usage constitutes acquisition of practical experience with HPC?”

### 4.5 Value added to the US economy by XSEDE

For 2019, the most recent year for which data are available, the average starting salary for non-computer-science STEM bachelor's degree recipients was $77,874; for new recipients of graduate degrees, it was $96,178 (Day and Martinez, 2021).

Considering Table 10 as a whole, it seems reasonable to take 1,500 as an approximate number of undergraduate students who acquired a practical level of experience using HPC resources, and 6,000 as the approximate number of graduate students who acquired such practical experience. These numbers are within the range of a set of seemingly reasonable definitions and in particular are very close to the number of undergraduate and graduate students who ran HPC jobs on a number of days that corresponds to a typical private sector work month (20 days).

**Table 10:** Potential definitions of “practical experience” and students using XSEDE-supported HPC resources.

**Practical experience**	**Undergraduate students**	**Undergraduates with such experience**	**Graduate students**	**Graduates with such experience**
Median days active	7 days	3,258	10 days	8,016
One work month	20 days	1,638	20 days	5,927
Median jobs run	29 jobs	1,120	49 jobs	8,195

To estimate the economic impact on the US economy generated by XSEDE training and XSEDE-supported HPC resources, we multiplied the estimated numbers for undergraduate (1,500) and graduate students (6,000) times a number of possible raise figures, as shown in Table 11. This table shows total and annualized estimates for the six years of operation of XSEDE2—2016 through 2022.

**Table 11:** Estimated economic impact due to HPC skills gained through XSEDE for 2016–2022^*^.

**Measure of raise**	**Value of raise**	**Total**	**Annually**
**Mean of all organizations**	**7.64%**	**$53,012,356**	**$8,835,393**
Mean of organizations that use HPC	15.2%	$105,469,608	$17,578,268
Median of all organizations	5%	$34,693,950	$5,782,325
Assume 10% differential	10%	$69,387,900	$11,564,650

^*^Estimated 1,500 undergraduate and 6,000 graduate students with HPC skills.

## 5 Discussion

The initial questions this paper poses are from the standpoint of organizations that hire STEM professionals and those analyzing the job market for STEM professionals. Is there evidence of a particular need for individuals with skills in HPC? And how much additional salary are employers willing to offer to new degree holders who have practical experience in HPC programming as compared to those who do not?

The answer to these questions is clear and unambiguous among the hiring managers surveyed as part of this study. The general shortage of STEM professionals in the US is felt particularly strongly within organizations that make use of HPC. The organizations surveyed in this study were predominantly from the private sector, so this is a particularly important need relative to the US economy. Both the qualitative evidence and the quantitative survey responses indicate that there is a great need for STEM professionals with HPC skills, that such people can be hard to find, and that STEM professionals who finish a degree and have education in and/or practical experience with HPC are particularly desired and preferentially hired. The qualitative responses from the survey in particular emphasize the value of knowledge of HPC, experience, education, inquisitiveness and curiosity described with a variety of terms, as well as other factors to be expected in any team work environment (such as flexibility and the ability to work well in groups).

Amid all the hype over AI and the strong trend toward using cloud computing, it is important to emphasize that the demand identified in our interviews and surveys is traditional HPC: clusters, parallel computing software, and supercomputers; i.e., the sorts of computing resources that the NSF has made available without direct financial cost to researchers and students since the 1980s (Stewart et al., 2019). While it was somewhat challenging for students to get accounts on the systems provided by the first generation of NSF supercomputer centers, due to the high demand relative to supply, there has been an increasing focus on providing resources for students since that time. Over the 11 years of operation of these facilities funded by the NSF, the XSEDE program provided training and access to HPC resources for tens of thousands of students (XSEDE, 2023, 2016).

This study has conclusively documented that employers are willing to offer higher starting salaries to recent graduates if they have HPC experience. Just over half of the hiring managers interviewed indicated that initial salary offers would be higher for new degree holders, at both the graduate and undergraduate level, who have practical experience in HPC programming than for those who do not.

We can provide a number of estimates of the percentage salary increase that a new graduate might expect as a result of having practical experience with HPC when they graduate. The best estimates of an overall range of impact on salary seems to be somewhere between 7% and 15%, representing the mean raise at all companies surveyed and the mean at the companies that indicated use of HPC. While the companies are not a random sample, they are a large sample of the companies that Intersect360 normally surveys in their analyses of the advanced computing market, and it is Intersect360's business proposition that their surveys offer a good representation of the market of organizations that make use of advanced computing. If we want to boil the data down to one overall estimate of expected impact, we can take the round figure of a 10% salary differential. For the new graduate taking a first job, this salary differential seems large enough to be financially significant, particularly when acquiring HPC experience can be done typically through taking no-cost or low-cost tutorials and seminars. A difference of 10% or even as low as 5% may be financially significant, particularly because this becomes effectively an annuity that pays off over their career as their salary is incrementally adjusted upward each year (barring some dramatic and unexpected event).

From the standpoint of a student considering undergraduate or graduate studies in a STEM field, we can offer several observations. First, there is clear demand for students who have a STEM degree and experience with HPC that exceeds the general and well-documented demand for students with a STEM focus. Furthermore, particularly for graduate students, the literature is very clear that research papers published based on use of HPC are cited more often than similar papers that do not make use of HPC resources (von Laszewski et al., 2015), suggesting that those graduate students who make use of HPC in their thesis or dissertation research may increase their chances of eventually obtaining a permanent academic degree. We do not know what particular benefits were obtained by the particular students who gained experience with HPC through XSEDE training programs and the opportunity to make use of XSEDE-supported HPC resources. We know for certain that students report a great deal of satisfaction with this program (Miles et al., 2023). We have good reason to believe that the many thousands of such students who were able to gain good education about and experience with HPC may have had a competitive advantage in seeking satisfying employment and may have received a higher salary as a result of this experience.

What value did XSEDE provide to the US economy? In the terms of an international accounting framework for assessing value created by organizations, “human capital” refers to “people's competencies, capabilities and experience, and their motivations to innovate” (The International Integrated Reporting Council (IIRC), 2021). We can estimate the value of the human capital that the XSEDE project created for the US as a whole during the time frame for which we have good student usage data - 2016 to 2022. Calculations based on several possible definitions are shown in Table 10 ranging from a total of 1,120–3,258 undergraduates and 5,927–8,016 graduate students. We calculated economic benefit using a somewhat conservative middle ground of 1,500 undergraduate students total and 6,000 graduate students total. Assuming, conservatively, that new hires without HPC experience would catch up in their skills within a year, and thus estimating the impact of XSEDE training to last for one year after a person is hired, we can estimate the value created by XSEDE through its provisioning of HPC resources. Using these figures and assumptions, human capital created by XSEDE is around $10 million per year. This value creation constitutes just under 20% of the total annualized cost of XSEDE and associated HPC systems. (The total average annualized cost for XSEDE and the HPC systems it supported was $51,416,624 (Snapp-Childs et al., 2024). A significant fraction of the students who made use of XSEDE were from EPSCoR states, where colleges and universities receive a disproportionately low percentage of total NSF grant awards (Miles et al., 2023). (EPSCoR is the name of an NSF program to aid grant competitiveness in such states; the abbreviation is short for Established Program to Stimulate Competitive Research). There are fewer HPC resources local to universities and colleges in these states, so access to national resources such as provided by XSEDE is particularly important for students in EPSCoR states. Human capital is one of many forms of value created by XSEDE that, as shown in Snapp-Childs et al. (2024), result in a net benefit to the US and its citizens that is greater than the total cost to the US government of XSEDE and the associated HPC systems.

The data presented here are extremely relevant to education and career planning for people considering a STEM major at the undergraduate or graduate level. For those pondering the pursuit of a bachelor's degree in a STEM field, the message is simple. Acquiring practical experience in using HPC during one's academic studies will make it possible to enter the job market with skills that are in great demand. Added to your undergraduate diploma, possession of HPC skills will make you part of a group of people highly sought after by potential employers. HPC might potentially result in a starting salary boost of as much as 7% to 15% or even more, an increase that becomes part of one's base salary and is thus an annuity that pays off each year, as that salary increases incrementally throughout a career.

For people considering a PhD, the data presented here are extremely interesting. People pursuing a PhD and who acquire HPC skills open new possibilities in their research which may lead to their published papers having a larger number of citations. Studies on citations of papers have consistently shown that peer-reviewed publications based on use of HPC as a research tool are cited more often than are similar papers not involving use of HPC (von Laszewski et al., 2015). This means that use of HPC in one's PhD research may aid the pursuit of a permanent academic position. But even with the use of HPC, only a minority of the people receiving a PhD in a particular area in any given year will probably obtain such a position. For the remainder who have acquired practical experience in HPC, the value proposition nevertheless still appears quite promising. A new PhD holder has had the opportunity to pursue their research passion and make a significant contribution to the knowledge of humankind, and will now hold the prestigious title of “Dr.” for life. For those new PhD holders who do not end up in a permanent academic position, the possession of HPC skills may give them a better chance of avoiding some of the problems and pitfalls identified in Sarrico (2022). People getting a PhD and at the same time gaining practical experience with HPC possess the potential to enter the job market with highly sought-after skills and a higher salary than would otherwise be the case.

Individuals who possess the necessary academic preparation to become STEM professionals often pursue further education, particularly education at the level of terminal degrees, based on their academic discipline preferences and intellectual passions, rather than solely considering the job market. It is important to fill such positions with the very most qualified individuals because of the relative permanence of, for example, tenured professor positions. Those selected for such positions influence the quality of higher education and research in the US for decades. A quick check of the personal recollections of most academics will reveal a general belief that things like GRE (Graduate Record Examination) scores are a poor predictor of career success as a PhD. This has been clearly documented for example in the field of biomedical research (Sealy et al., 2019). What this means is that only upon completion of the PhD, with the results of a person's research in hand, can one judge relative merit for top academic jobs. Having the best possible group of PhD holders leading research and education in US universities and colleges cannot be achieved by trying to match the total number of students accepted into graduate schools nationwide with the total number of jobs to be filled in each particular area. What is required is an overproduction of PhDs so that the best can then be identified on the basis of their research innovations and accomplishments (along with other factors). An overproduction of new PhDs relative to the needs of the academic market is thus essential for the high quality of the US education system and US global competitiveness. Without some excess production of new PhDs relative to the permanent positions available, there would be no competition and no ability to ensure that the very best new PhD holders were the ones moving into tenured (or other permanent) academic roles.

The data presented here suggest that those contemplating a graduate degree in a STEM field have a solid value proposition for their education path. This is true even if they ultimately do not secure a permanent academic position, such as a tenured professorship, as their graduate education still effectively translates into a viable career. A shift over time of new PhDs moving into roles other than those within academia has been documented in a Forbes article (Nietzel, 2021) that states, “In 2000, 48.6% of those earning their doctorate took employment in academia; by 2020 that percentage had dropped to 39.6%. On the other hand, 26% of doctoral recipients were initially employed in business and industry in 2000, but by 2020 that percentage had jumped to 40%.... People with PhDs are more and more entering job markets other than the ranks of academia.” What the data presented here suggest is that those who do not end up in a permanent position in academia have - all else being equal - a better chance of finding a job and a good chance of a higher salary if they acquire practical experience with HPC as part of their graduate studies. This viewpoint is consistent with another recent discussion of how lab leaders can aid students in their lab who will move outside of academia as part of their career path (Forrester, 2022).

The findings here also match the personal experiences of the authors, a majority of whom have graduate degrees in a STEM field other than computer science and nevertheless have careers in advanced computing. Indeed, one of the authors (CAS) made it a practice for many years to hire people who held recent PhDs in astronomy as supercomputer administrators and programmers. This was because, while there are far more astronomers completing PhDs than there are available positions for astronomers in academia and private research, the training required for completing a PhD in astronomy generally builds the skills that enable such people to be excellent professionals working in advanced computing system operations or support. The personal experiences of the authors of this paper are just one window into a vast ecosystem of innovation in which some people acquire permanent (often tenured) positions in academia, and others - many of them by no means less qualified - pursue meaningful and valuable careers through other mechanisms.

The annual economic impact of the training and educational opportunities provided by XSEDE estimated here amounts to a total of around $10 million per year of operation of the 2016–2022 phase of the XSEDE project (referred to as XSEDE2 within the NSF). This $10 million figure is within a set of different estimates that ranges, depending on what assumptions are made and which figures are used, from a low of slightly over $5 million per year to around $17 million, and is probably a reasonable “middle ground” estimate of economic value created through training activities. Because the data we collected are not a random sample of any particular population but rather a sample obtained with the intent of getting good coverage of organizations that were perceived to be likely to hire people with HPC experience, we are not able to present here any statistically derived measures of confidence intervals.

This economic benefit in the form of “human capital” is just one of the many types of value created by a support and operations project that operated with an annual budget of between $20 and $25 million per year over the time period studied, and it is notable even in comparison to the total approximately $60 million annualized cost of XSEDE and the several HPC resources it supported. This contribution to human capital is one of many factors that suggests that the US as a whole received more benefit from taxpayer investment in XSEDE and the HPC resources supported by XSEDE than these investments cost the US federal government (Snapp-Childs et al., 2024).

The results of this study suggest that from the standpoint of the US economy, investments at the federal level in XSEDE and its successor, the ACCESS project (Boerner et al., 2023), have had a potentially important impact on the critical supply of STEM workers with HPC experience within the US. Given current dependence upon STEM workers born outside the US, there is a great opportunity to help the US economy and provide excellent career prospects for those born in the US. This sort of practical experience is also provided at many universities and colleges with on-premise HPC systems; however, the US federal government and many state governments are chronically under pressure. Within the last year, three major US universities announced budget shortfalls in the $10s of millions [detailed in Snapp-Childs et al. (2024)]. This spring, a dozen other smaller colleges and universities announced major budget and program cuts (Moody, 2024). As organizations from the US government down to small colleges seek to optimize their budgets, this study suggests that investments in facilities that provide students with opportunities to obtain practical competence with HPC are very important. Providing such facilities on their premises is beyond the budget possibilities of many individual colleges and universities. Federal programs such as XSEDE and ACCESS are important because they provide access to HPC systems for students at any institution of higher education in the US.

The findings here also provide potentially useful information for those planning and executing credit-bearing and non-credit-bearing educational activities at the undergraduate and graduate levels. As clearly indicated in reports that rank colleges and universities, the relationship between a higher education degree and employment and careers is an important factor in the minds of students and graduates (Corrigan and Mcallister, 2024). The availability of access to NSF-funded HPC resources and of practical ways to provide local resources even at institutions with limited funding means that this sort of educational opportunity should be within the reach of any institution willing and able to fund well-qualified and effective instructors (Boerner et al., 2023; XSEDE, 2023; Chakravorty et al., 2024).

Three observations should be made regarding the data we collected and present here. First, these kinds of data are difficult to obtain because they may easily reveal competitively sensitive information. As a result, it's difficult to get such information through standard survey methods. Second, because of this, we used a trusted private sector market analysis firm to obtain data from a suite of organizations that are representative of STEM-oriented organizations generally, rather than some sort of random sample. This approach enabled us to get good, actionable information while also suggesting possible venues for further more detailed research. There is also a reasonable amount of scatter that we believe is attributable to relatively sparse sampling along the joint dimensions of organizational size and market sector. The recent paper by Lysenko and Wang (2023) shows how many sometimes subtle factors influence job-seeking success on the part of new graduates and hiring practices on the part of organizations. Drawing from their example and given the signals present in the data we collected, a much larger study on the topics addressed here seems useful. Third, and perhaps most importantly, despite the limitations in our dataset, the data support our conclusions. These findings are strong enough to guide practical and operational decision-making by potential students, current students, educational organizations, and governmental agencies responsible for policy-making and funding related to research and education. New degree holders with HPC practical experience may not be able to just enter the job market and receive a higher starting salary as an automatic result of possessing these skills. However, it's clear that HPC skills enhance one's employability and may, with a bit of selective attention on the part of job seekers, also result in an increased starting salary.

The research and discussion presented here are very much US-focused. However, the shortage of STEM workers and overproduction of PhDs in certain academic areas are phenomena that impact many nations. A recent report about the European Union reads very much like similar reports for the US (European Commission, 2023). Another report paints a similar, but perhaps more dire, picture for Africa (Amegah et al., 2023). The core educational strategy suggested here - that students in STEM fields, particularly doctoral students in disciplines where there may be an oversupply of new PhDs relative to academic market demands, can and should be provided with opportunities to gain HPC experience that will make them valuable knowledge workers and give them better-paid career flexibility and security—ought to be applicable in any industrialized or developing nation. Indeed, to the extent that many students from such nations pursue advanced degrees in the US, there is at least a possibility for such students to get a degree in the US, gain some work experience here, and then return to aid the advancement of their home countries. There are also potential implications for universities that operate internationally. Such universities provide many opportunities for students across the globe, but as regards US universities in particular, these undertakings involve at least two potential moral hazards: first, that a student might receive an education but then be unable to pursue a fulfilling career; and second, that providing higher education degrees in developing countries will result in the transport of talent from those countries to the US. For universities operating internationally and based in the US, the findings reported here suggest that both moral hazards could be mitigated by including education and practical experiences with HPC. Students taking advantage of such learning opportunities would certainly have access to good job opportunities and possibly, at least, the opportunity to work in and benefit their homeland.

## 6 Conclusion

In terms of the job market, the data presented here indicate that the widely recognized shortage of STEM professionals in the US is particularly pronounced for organizations that depend heavily on HPC technology. Given that a significant number of HPC users are high-tech US manufacturing firms, professionals with STEM backgrounds and HPC skills are particularly important to the US economy. Their expertise is especially relevant to market sectors associated with the US's global competitiveness and national defense. The shortage of STEM workers with HPC skills relative to demand creates an economic bonus for the possession of such skills that, according to the organizations we surveyed, amounts to a roughly 10% starting salary increase for new hires having recently completed a degree and acquired practical experience using HPC in the process.

In this paper, we have demonstrated that from the standpoint of any individual considering a major in a STEM field at any level of post-secondary education, there is great value in obtaining practical experience with the use of HPC facilities. For those pursuing a doctoral degree, in particular, there is clear evidence that doctoral research that makes use of HPC resources is more highly cited than similar research that does not make use of HPC resources, suggesting that those who incorporate HPC-based techniques may have an advantage, all else being equal, in competing for permanent jobs in academia. Meanwhile, for those who receive a doctoral degree and do not find themselves on the way to a permanent academic position, practical experience with HPC may lead to opportunities for a career based on STEM and HPC that is associated with higher-than-average remuneration and high demand as a candidate in today's job market.

From the standpoint of those investing in HPC facilities, particularly decision-makers in the federal government and institutions in higher education, there is tremendous value in providing resources and support for students to acquire practical experience in the use of HPC. As a particular example, XSEDE played a significant role in generating human capital equivalent to half of the annual total cost of XSEDE to the US federal government. This factor, among others previously discussed, further supports the notion that XSEDE has proven to be a particularly effective investment of US federal government research and development funds.

The results presented here suggest a way to reconcile individual intellectual passions with the needs of the STEM job market and national competitiveness. There is considerable value to the individual in the ability to pursue their intellectual interests to as great an extent as they care to, up to and including a terminal degree. Many people pursue their educational programs out of interest and passion rather than their reading of job market needs. Plus, someone has to get those tenure-track jobs; each new graduate student has a right to believe, “it might as well be me.” From the standpoint of the country as a whole, producing more PhDs than the academic faculty market requires ensures that competition leads to the best possible academic faculty leading research and educating future generations of students. If a consequence of letting as many qualified people as possible pursue their intellectual passions is an oversupply of new degree holders in a particular area this can still be good both for individuals and the nation - so long as students also acquire some marketable skill in areas of high demand. We have demonstrated that the acquisition of HPC skills places newly graduated students at all levels into a group that is in high demand in the market, meaning that such students should have an easier time finding a good job overall and students with HPC skills have an opportunity to get a higher starting salary than others.

Educational facilities that enable students to obtain practical HPC experience are important to the future of the US economy *and* to the best interests of students. Those universities and colleges that are able to invest in local facilities should place a high priority on doing so. It is particularly important for the US federal government to prioritize the provision of access to HPC facilities at a national level, so that all higher education students in the US have the opportunity to acquire competence in HPC. Federal investment in the XSEDE project, through the NSF, has provided significant economic value to the US in many ways. This paper shows that one important part of the value created by XSEDE is human capital. This is in the form of people who gained HPC skills through XSEDE training programs and use of XSEDE resources. This is an aspect of the federal return on investment in XSEDE that will pay dividends for decades to come.

This paper has presented data of a sort that, to our knowledge, has never been collected and openly published before. It offers new metrics for the value of practical HPC experience and market needs in the US for professionals with HPC expertise. This work also particularly builds on and adds new information to the analyses created by Lysenko and Wang (2023), which aim, among other things, to create a model of employment expectations (chances of being hired and expected salary) for STEM graduates. Their model covers many aspects but does not include the possession or lack of HPC experience. The work presented here suggests that this and likely other factors can be added to their work and expand their valuable contribution. We believe and hope that the information presented here will be of use to both individuals and institutions within the US, and that this research will lead to further empirical research on the value to individual workers and to the US as a whole of a STEM workforce competent in the use of HPC.

## Data Availability

The datasets presented in this study can be found in online repositories. The names of the repository/repositories and accession number(s) can be found in Snapp-Childs et al. (2023a,b).
